# 2-(4-Bromo­phen­yl)-2-oxoethyl 4-methyl­benzoate

**DOI:** 10.1107/S1600536811045272

**Published:** 2011-11-02

**Authors:** Hoong-Kun Fun, Tara Shahani, B. Garudachari, Arun M. Isloor, M. N. Satyanarayan

**Affiliations:** a X-ray Crystallography Unit, School of Physics, Universiti Sains Malaysia, 11800 USM, Penang, Malaysia; b Medicinal Chemistry Division, Department of Chemistry, National Institute of Technology-Karnataka, Surathkal, Mangalore 575 025, India; c Department of Physics, National Institute of Technology-Karnataka, Surathkal, Mangalore 575 025, India

## Abstract

The title compound, C_16_H_13_BrO_3_, consists of a toluene ring and a bromo­benzene ring which are linked together by a 2-oxopropyl acetate group. The dihedral angle formed between the toluene and bromo­benzene rings is 80.70 (7)°. In the crystal, inter­molecular C—H⋯O hydrogen bonds link the mol­ecules into a three-dimensional network.

## Related literature

For applications of phenacyl benzoate derivatives, see: Rather & Reid (1919 ▶); Judefind & Reid (1920 ▶); Gandhi *et al.* (1995 ▶); Huang *et al.* (1996 ▶); Sheehan & Umezaw (1973 ▶); Ruzicka *et al.* (2002 ▶); Litera *et al.* (2006 ▶). For a related structure, see: Fun *et al.* (2011 ▶). For bond-length data, see: Allen *et al.* (1987 ▶).
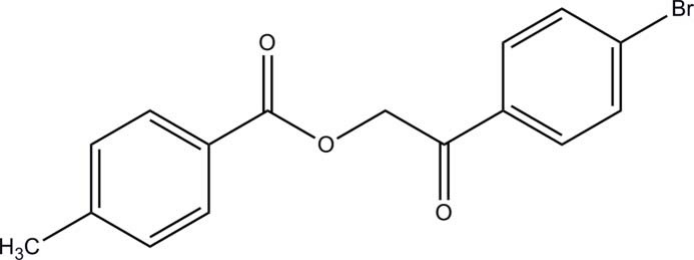


## Experimental

### 

#### Crystal data

C_16_H_13_BrO_3_
*M* *_r_* = 333.17
Monoclinic, 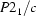

*a* = 5.8368 (2) Å
*b* = 8.3438 (3) Å
*c* = 27.9684 (8) Å
β = 95.177 (1)°
*V* = 1356.54 (8) Å^3^
*Z* = 4
Mo *K*α radiation
μ = 3.03 mm^−1^
*T* = 100 K
0.50 × 0.14 × 0.12 mm

#### Data collection

Bruker SMART APEXII CCD area-detector diffractometer
Absorption correction: multi-scan (*SADABS*; Bruker, 2009 ▶) *T*_min_ = 0.311, *T*_max_ = 0.707
14735 measured reflections
3936 independent reflections
3395 reflections with *I* > 2σ(*I*)
*R*_int_ = 0.026

#### Refinement

*R*[*F* ^2^ > 2σ(*F* ^2^)] = 0.028
*wR*(*F* ^2^) = 0.074
*S* = 1.02
3936 reflections
182 parameters
H-atom parameters constrained
Δρ_max_ = 0.45 e Å^−3^
Δρ_min_ = −0.42 e Å^−3^

### 

Data collection: *APEX2* (Bruker, 2009 ▶); cell refinement: *SAINT* (Bruker, 2009 ▶); data reduction: *SAINT*; program(s) used to solve structure: *SHELXTL* (Sheldrick, 2008 ▶); program(s) used to refine structure: *SHELXTL*; molecular graphics: *SHELXTL*; software used to prepare material for publication: *SHELXTL* and *PLATON* (Spek, 2009 ▶).

## Supplementary Material

Crystal structure: contains datablock(s) global, I. DOI: 10.1107/S1600536811045272/hg5125sup1.cif

Structure factors: contains datablock(s) I. DOI: 10.1107/S1600536811045272/hg5125Isup2.hkl

Supplementary material file. DOI: 10.1107/S1600536811045272/hg5125Isup3.cml

Additional supplementary materials:  crystallographic information; 3D view; checkCIF report

## Figures and Tables

**Table 1:** Hydrogen-bond geometry (Å, °)

*D*—H⋯*A*	*D*—H	H⋯*A*	*D*⋯*A*	*D*—H⋯*A*
C16—H16*A*⋯O2^i^	0.98	2.42	3.355 (2)	160
C16—H16*B*⋯O3^ii^	0.98	2.53	3.451 (2)	157

Symmetry codes: (i) 
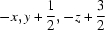
; (ii) 
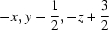
.

**Fig. 1.**
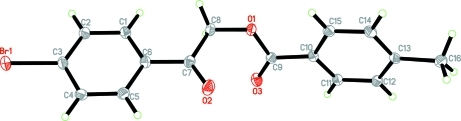
The molecular structure of the title compound, showing 30% probability displacement ellipsoids and the atom-numbering scheme.

**Fig. 2.**
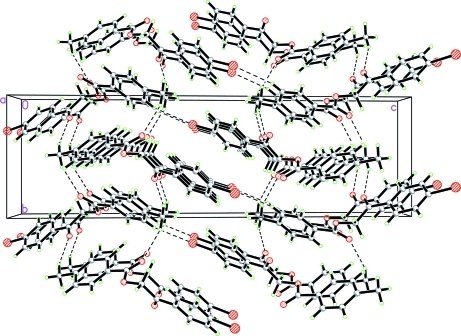
The crystal packing of the title compound, viewed along a axis. Intermolecular hydrogen bonds linked the molecules into three-dimensional network.

## supplementary crystallographic information

### Comment

In organic chemistry, phenacyl benzoate is a derivative of an acid which is formed by the reaction between an acid and a phenacyl bromide. They find applications in the field of synthetic chemistry (Rather & Reid, 1919; Huang *et al.*, 1996; Gandhi *et al.*, 1995) such as in the synthesis of oxazoles, imidazoles, benzoxazepines. They are also useful as photo-removable protecting groups for carboxylic acids in organic synthesis and biochemistry (Ruzicka *et al.*, 2002; Litera *et al.*, 2006; Sheehan & Umezaw, 1973). Keeping this in view, the title compound was synthesized to study its crystal structure.

The title compound, (Fig. 1), consists of a toluene ring (C10–C16) and a bromobenzene ring (C1–C6/Br1) which are linked together by a 2-oxopropyl acetate group (C7–C9/O1–O3). The dihedral angle formed between the toluene and bromobenzene rings is 80.70 (7)%. The bond lengths (Allen *et al.*, 1987) and angles are within normal ranges and are comparable to these closely related structure (Fun *et al.*, 2011).

In the crystal packing (Fig. 2), intermolecular C16—H16A···O2 and C16—H16B···O3 hydrogen bonds (Table 1) link the molecules into a three-dimensional network.

### Experimental

The mixture of 4-methylbenzoic acid (1.0 g, 0.0073 mol), potassium carbonate (1.10 g, 0.0080 mol) and 2-bromo-1-(4-bromophenyl)ethanone (2.02 g, 0.0073 mol) in dimethylformamide (10 ml) was stirred at room temperature for 2 h. On cooling, the separated yellow needle-shaped crystals of 2-(4-bromophenyl)-2-oxoethyl 4-methylbenzoate were collected by filtration. Compound was recrystallized from ethanol. Yield: 2.22 g, 90.98%. M.p.: 425–426 K (Judefind & Reid, 1920).

### Refinement

All the H atoms were positioned geometrically [C–H = 0.9500–0.9900 Å] and were refined using a riding model, with *U*_iso_(H) = 1.2 or 1.5*U*_iso_(C).

### Crystal data

**Table:**

C_16_H_13_BrO_3_	*F*(000) = 672
*M**_r_* = 333.17	*D*_x_ = 1.631 Mg m^−^^3^
Monoclinic, *P*2_1_/*c*	Mo *K*α radiation, λ = 0.71073 Å
Hall symbol: -P 2ybc	Cell parameters from 6134 reflections
*a* = 5.8368 (2) Å	θ = 2.6–32.4°
*b* = 8.3438 (3) Å	µ = 3.03 mm^−^^1^
*c* = 27.9684 (8) Å	*T* = 100 K
β = 95.177 (1)°	Needle, yellow
*V* = 1356.54 (8) Å^3^	0.50 × 0.14 × 0.12 mm
*Z* = 4	

### Data collection

**Table:**

Bruker SMART APEXII CCD area-detector diffractometer	3936 independent reflections
Radiation source: fine-focus sealed tube	3395 reflections with *I* > 2σ(*I*)
graphite	*R*_int_ = 0.026
φ and ω scans	θ_max_ = 30.0°, θ_min_ = 1.5°
Absorption correction: multi-scan (*SADABS*; Bruker, 2009)	*h* = −8→8
*T*_min_ = 0.311, *T*_max_ = 0.707	*k* = −11→11
14735 measured reflections	*l* = −39→39

### Refinement

**Table:**

Refinement on *F*^2^	Primary atom site location: structure-invariant direct methods
Least-squares matrix: full	Secondary atom site location: difference Fourier map
*R*[*F*^2^ > 2σ(*F*^2^)] = 0.028	Hydrogen site location: inferred from neighbouring sites
*wR*(*F*^2^) = 0.074	H-atom parameters constrained
*S* = 1.02	*w* = 1/[σ^2^(*F*_o_^2^) + (0.0324*P*)^2^ + 1.2242*P*] where *P* = (*F*_o_^2^ + 2*F*_c_^2^)/3
3936 reflections	(Δ/σ)_max_ = 0.002
182 parameters	Δρ_max_ = 0.45 e Å^−^^3^
0 restraints	Δρ_min_ = −0.42 e Å^−^^3^

### Special details

**Table:**

Geometry. All e.s.d.'s (except the e.s.d. in the dihedral angle between two l.s. planes) are estimated using the full covariance matrix. The cell e.s.d.'s are taken into account individually in the estimation of e.s.d.'s in distances, angles and torsion angles; correlations between e.s.d.'s in cell parameters are only used when they are defined by crystal symmetry. An approximate (isotropic) treatment of cell e.s.d.'s is used for estimating e.s.d.'s involving l.s. planes.
Refinement. Refinement of *F*^2^ against ALL reflections. The weighted *R*-factor *wR* and goodness of fit *S* are based on *F*^2^, conventional *R*-factors *R* are based on *F*, with *F* set to zero for negative *F*^2^. The threshold expression of *F*^2^ > σ(*F*^2^) is used only for calculating *R*-factors(gt) *etc*. and is not relevant to the choice of reflections for refinement. *R*-factors based on *F*^2^ are statistically about twice as large as those based on *F*, and *R*- factors based on ALL data will be even larger.

### Fractional atomic coordinates and isotropic or equivalent isotropic displacement parameters (Å^2^)

**Table:**

	*x*	*y*	*z*	*U*_iso_*/*U*_eq_	
Br1	1.36051 (3)	0.73030 (2)	1.049704 (6)	0.02370 (7)	
O1	0.6371 (2)	1.02498 (16)	0.79328 (4)	0.0188 (3)	
O2	0.5266 (2)	0.82885 (16)	0.86329 (5)	0.0228 (3)	
O3	0.3804 (2)	1.16704 (15)	0.83172 (4)	0.0193 (3)	
C1	1.0857 (3)	0.9333 (2)	0.92209 (6)	0.0166 (3)	
H1A	1.1334	1.0088	0.8996	0.020*	
C2	1.2317 (3)	0.8944 (2)	0.96256 (6)	0.0177 (3)	
H2A	1.3795	0.9423	0.9677	0.021*	
C3	1.1589 (3)	0.7853 (2)	0.99509 (6)	0.0171 (3)	
C4	0.9420 (3)	0.7134 (2)	0.98883 (6)	0.0184 (3)	
H4A	0.8935	0.6401	1.0119	0.022*	
C5	0.7995 (3)	0.7520 (2)	0.94806 (7)	0.0179 (3)	
H5A	0.6523	0.7032	0.9429	0.022*	
C6	0.8693 (3)	0.86164 (19)	0.91454 (6)	0.0154 (3)	
C7	0.7111 (3)	0.8959 (2)	0.87074 (6)	0.0161 (3)	
C8	0.7881 (3)	1.0191 (2)	0.83609 (6)	0.0177 (3)	
H8A	0.7936	1.1258	0.8516	0.021*	
H8B	0.9453	0.9925	0.8279	0.021*	
C9	0.4324 (3)	1.09906 (19)	0.79604 (6)	0.0150 (3)	
C10	0.2867 (3)	1.08669 (19)	0.74980 (6)	0.0146 (3)	
C11	0.0783 (3)	1.1700 (2)	0.74432 (6)	0.0161 (3)	
H11A	0.0369	1.2395	0.7690	0.019*	
C12	−0.0687 (3)	1.1510 (2)	0.70261 (6)	0.0173 (3)	
H12A	−0.2101	1.2081	0.6991	0.021*	
C13	−0.0108 (3)	1.0493 (2)	0.66597 (6)	0.0155 (3)	
C14	0.2036 (3)	0.9726 (2)	0.67110 (6)	0.0168 (3)	
H14A	0.2490	0.9076	0.6457	0.020*	
C15	0.3505 (3)	0.9897 (2)	0.71243 (6)	0.0166 (3)	
H15A	0.4943	0.9357	0.7154	0.020*	
C16	−0.1776 (3)	1.0169 (2)	0.62159 (6)	0.0178 (3)	
H16A	−0.2907	1.1038	0.6176	0.027*	
H16B	−0.2572	0.9150	0.6256	0.027*	
H16C	−0.0917	1.0111	0.5931	0.027*	

### Atomic displacement parameters (Å^2^)

**Table:**

	*U*^11^	*U*^22^	*U*^33^	*U*^12^	*U*^13^	*U*^23^
Br1	0.02204 (10)	0.03193 (11)	0.01642 (9)	0.00268 (7)	−0.00216 (6)	0.00657 (7)
O1	0.0149 (6)	0.0279 (6)	0.0133 (6)	0.0042 (5)	−0.0009 (5)	0.0007 (5)
O2	0.0189 (6)	0.0216 (6)	0.0267 (7)	−0.0048 (5)	−0.0049 (5)	0.0034 (5)
O3	0.0223 (6)	0.0201 (6)	0.0151 (6)	0.0030 (5)	0.0003 (5)	−0.0024 (5)
C1	0.0174 (8)	0.0179 (7)	0.0143 (8)	−0.0008 (6)	0.0001 (6)	0.0017 (6)
C2	0.0166 (8)	0.0202 (8)	0.0159 (8)	−0.0012 (6)	0.0000 (6)	−0.0002 (6)
C3	0.0179 (8)	0.0201 (8)	0.0130 (7)	0.0040 (6)	0.0006 (6)	0.0005 (6)
C4	0.0183 (8)	0.0190 (8)	0.0183 (8)	0.0017 (6)	0.0045 (6)	0.0039 (6)
C5	0.0153 (8)	0.0177 (8)	0.0207 (8)	0.0006 (6)	0.0014 (6)	0.0017 (6)
C6	0.0168 (8)	0.0142 (7)	0.0148 (7)	0.0009 (6)	−0.0003 (6)	−0.0008 (6)
C7	0.0166 (8)	0.0152 (7)	0.0160 (8)	0.0020 (6)	−0.0005 (6)	−0.0011 (6)
C8	0.0155 (8)	0.0220 (8)	0.0150 (8)	−0.0003 (6)	−0.0025 (6)	0.0022 (6)
C9	0.0157 (7)	0.0138 (7)	0.0154 (8)	−0.0005 (6)	0.0007 (6)	0.0022 (6)
C10	0.0154 (7)	0.0146 (7)	0.0136 (7)	−0.0004 (6)	0.0012 (6)	0.0011 (6)
C11	0.0168 (8)	0.0177 (7)	0.0139 (7)	0.0011 (6)	0.0014 (6)	−0.0003 (6)
C12	0.0153 (8)	0.0188 (8)	0.0176 (8)	0.0031 (6)	0.0006 (6)	0.0023 (6)
C13	0.0167 (8)	0.0164 (7)	0.0134 (7)	−0.0033 (6)	0.0020 (6)	0.0036 (6)
C14	0.0204 (8)	0.0154 (7)	0.0151 (8)	−0.0001 (6)	0.0036 (6)	−0.0018 (6)
C15	0.0162 (8)	0.0163 (7)	0.0175 (8)	0.0018 (6)	0.0016 (6)	0.0005 (6)
C16	0.0164 (8)	0.0174 (7)	0.0198 (8)	0.0002 (6)	0.0018 (6)	0.0033 (6)

### Geometric parameters (Å, °)

**Table:**

Br1—C3	1.8986 (16)	C8—H8A	0.9900
O1—C9	1.354 (2)	C8—H8B	0.9900
O1—C8	1.4222 (19)	C9—C10	1.486 (2)
O2—C7	1.215 (2)	C10—C11	1.397 (2)
O3—C9	1.210 (2)	C10—C15	1.399 (2)
C1—C2	1.393 (2)	C11—C12	1.394 (2)
C1—C6	1.396 (2)	C11—H11A	0.9500
C1—H1A	0.9500	C12—C13	1.395 (2)
C2—C3	1.381 (2)	C12—H12A	0.9500
C2—H2A	0.9500	C13—C14	1.402 (2)
C3—C4	1.397 (3)	C13—C16	1.531 (2)
C4—C5	1.387 (2)	C14—C15	1.383 (2)
C4—H4A	0.9500	C14—H14A	0.9500
C5—C6	1.397 (2)	C15—H15A	0.9500
C5—H5A	0.9500	C16—H16A	0.9800
C6—C7	1.494 (2)	C16—H16B	0.9800
C7—C8	1.509 (2)	C16—H16C	0.9800
			
C9—O1—C8	116.81 (14)	O3—C9—O1	123.32 (15)
C2—C1—C6	120.12 (16)	O3—C9—C10	125.74 (16)
C2—C1—H1A	119.9	O1—C9—C10	110.94 (14)
C6—C1—H1A	119.9	C11—C10—C15	119.59 (15)
C3—C2—C1	119.15 (16)	C11—C10—C9	119.01 (15)
C3—C2—H2A	120.4	C15—C10—C9	121.37 (15)
C1—C2—H2A	120.4	C12—C11—C10	119.97 (16)
C2—C3—C4	121.99 (16)	C12—C11—H11A	120.0
C2—C3—Br1	118.94 (13)	C10—C11—H11A	120.0
C4—C3—Br1	119.08 (13)	C11—C12—C13	120.80 (16)
C5—C4—C3	118.20 (16)	C11—C12—H12A	119.6
C5—C4—H4A	120.9	C13—C12—H12A	119.6
C3—C4—H4A	120.9	C12—C13—C14	118.42 (15)
C4—C5—C6	120.94 (16)	C12—C13—C16	121.62 (15)
C4—C5—H5A	119.5	C14—C13—C16	119.94 (15)
C6—C5—H5A	119.5	C15—C14—C13	121.25 (16)
C1—C6—C5	119.59 (15)	C15—C14—H14A	119.4
C1—C6—C7	121.73 (15)	C13—C14—H14A	119.4
C5—C6—C7	118.66 (15)	C14—C15—C10	119.84 (16)
O2—C7—C6	121.70 (16)	C14—C15—H15A	120.1
O2—C7—C8	120.97 (15)	C10—C15—H15A	120.1
C6—C7—C8	117.33 (14)	C13—C16—H16A	109.5
O1—C8—C7	111.48 (14)	C13—C16—H16B	109.5
O1—C8—H8A	109.3	H16A—C16—H16B	109.5
C7—C8—H8A	109.3	C13—C16—H16C	109.5
O1—C8—H8B	109.3	H16A—C16—H16C	109.5
C7—C8—H8B	109.3	H16B—C16—H16C	109.5
H8A—C8—H8B	108.0		
			
C6—C1—C2—C3	−0.5 (3)	C8—O1—C9—O3	4.6 (2)
C1—C2—C3—C4	−0.5 (3)	C8—O1—C9—C10	−176.21 (14)
C1—C2—C3—Br1	179.08 (13)	O3—C9—C10—C11	5.6 (3)
C2—C3—C4—C5	1.2 (3)	O1—C9—C10—C11	−173.61 (15)
Br1—C3—C4—C5	−178.36 (13)	O3—C9—C10—C15	−172.35 (17)
C3—C4—C5—C6	−1.0 (3)	O1—C9—C10—C15	8.5 (2)
C2—C1—C6—C5	0.7 (3)	C15—C10—C11—C12	2.6 (3)
C2—C1—C6—C7	−177.82 (16)	C9—C10—C11—C12	−175.38 (16)
C4—C5—C6—C1	0.1 (3)	C10—C11—C12—C13	0.1 (3)
C4—C5—C6—C7	178.61 (16)	C11—C12—C13—C14	−3.0 (3)
C1—C6—C7—O2	177.11 (17)	C11—C12—C13—C16	175.28 (16)
C5—C6—C7—O2	−1.4 (3)	C12—C13—C14—C15	3.4 (3)
C1—C6—C7—C8	−3.6 (2)	C16—C13—C14—C15	−174.92 (16)
C5—C6—C7—C8	177.94 (16)	C13—C14—C15—C10	−0.8 (3)
C9—O1—C8—C7	75.60 (19)	C11—C10—C15—C14	−2.2 (3)
O2—C7—C8—O1	−8.3 (2)	C9—C10—C15—C14	175.70 (16)
C6—C7—C8—O1	172.36 (14)		

### Hydrogen-bond geometry (Å, °)

**Table:**

*D*—H···*A*	*D*—H	H···*A*	*D*···*A*	*D*—H···*A*
C16—H16A···O2^i^	0.98	2.42	3.355 (2)	160.
C16—H16B···O3^ii^	0.98	2.53	3.451 (2)	157.

Symmetry codes: (i) −*x*, *y*+1/2, −*z*+3/2; (ii) −*x*, *y*−1/2, −*z*+3/2.
